# Perceived values and climate change resilience dataset in Siaya County, Kenya

**DOI:** 10.1016/j.dib.2024.110317

**Published:** 2024-03-12

**Authors:** Pu Yang, Alycia Leonard, Micaela Flores Lanza, Makena Ireri, Stephanie Hirmer

**Affiliations:** a Department of Engineering Science, University of Oxford, Oxford OX2 0ES, UK; b CLASP, Mezzanine Floor Rivaan Center, Muguga Green Road, Nairobi, Kenya

**Keywords:** Needs assessment, Value data, User perceived value, Beneficiaries, Developing countries, Sub-Saharan Africa

## Abstract

This dataset presents perceived values and socioeconomic indicators collected in Siaya, a rural county in Kenya in 2022. The data was obtained from 300 household surveys and group interviews conducted in six sub-counties across eleven villages. Socioeconomic data were collected with a special focus on climate change vulnerability. Information on housing, health, water accessibility and usage, electricity accessibility and usage, extreme weather events, community service, and information accessibility were mapped across survey questions. The user-perceived value (UPV) game – a perception-based surveying approach – was used to elicit local communities’ needs and perceptions of climate change challenges. The UPV game involves asking interviewees to select which graphically depicted items would be most necessary in different situations and probing them for the reasons behind their choices (why-probing). The data was collected in two languages (Dholuo and English) and then translated into English. These surveys and interviews were conducted to better understand the needs of rural Kenyan communities and their perceptions of climate change, with the aim to identify ways to build resilience. Kenyan policymakers can use the dataset to inform county-level energy and development plans, while researchers and development practitioners can use the dataset to better design their research and programmes to reflect local needs and values.

Specifications Table

**Table:** 

Subject	Social science
Specific subject area	Planning and development
Type of data	JavaScript Object Notation (JSON) file, Comma-separated values (CSV) file, accompanying tables and figures.
How the data were acquired	Data was collected through face-to-face interviews, using the user-perceived value (UPV) game, and a socio-economic household survey. Semi-structured survey data is documented in the CSV and JSON files. Descriptive statistics are presented in tables and figures. Interview data was obtained from UPV game transcripts.
Data format	Raw and analysed
Description of data collection	The data collection process involved targeted community sampling to recruit participants who represent different social and economic needs of residents in Siaya county. County coordinators recruited participants from venues like restaurants, places of worship, markets and community groups from within each community. Grassroots authorities, organizations, and societies were also approached to ensure the inclusion of marginalised groups, for example, the elderly, youth and people with disabilities. Consent forms were signed by all participants.
Data source location	Siaya (County of Kenya)
Data accessibility	Repository name: Zenodo Data identification number: 10.5281/zenodo.10160737 Direct URL to data: https://zenodo.org/records/10160737

## 1 Value of the Data

• The data can be used to understand what is important to rural communities in Kenya and to identify potential factors influencing decision-making for different demographic groups (e.g. women, youth, elderly and low-income).
• The rich, verbatim interview text sheds light on the perception of climate change and vulnerabilities within communities.
• Using the data to identify gaps in existing infrastructure and services, policymakers could determine where resources could be allocated to improve overall community well-being.
• This dataset can be used to identify the qualities comprising community resilience. It reports on multiple socioeconomic statuses relevant to rural Kenya, including; community networks and relationships; communication; health; governance and leadership; resources; preparedness; mental outlook; and economic investment.
• The role of energy services within community resilience can be examined in detail and used to enhance community resilience against climatic and social risks, and vulnerabilities.
• Academics can use the cleaned dataset directly for analysis, or it can be used to guide the collection of further sub-national data which reflects the needs of rural communities.

## 2 Objective

Sustainable development initiatives in the 21st century must increase well-being under the constraints of climate change. However, the perception of well-being among citizens themselves remains generally under-studied in developing regions. In order to develop effective strategies for reducing poverty and improving living standards in the face of climate change, surveys and interviews are required to provide information on what people value and need.

This dataset gathers insights from rural communities in Siaya County, Kenya regarding their values, needs, and perceptions of climate change challenges with a focus on energy services. This data can be used to identify ways to build community resilience and to understand the role of energy in climate change adaptation measures. The data was collected through a perception-based surveying approach called the user-perceived value (UPV) game. This involves interviewees selecting items which they value most from a contextualised set of graphically depicted items, and subsequently asking the reasons behind their selection (i.e., why-probing). For more details on the UPV methodology, please refer to Hirmer [1]. A socioeconomic survey was conducted to contextualize the UPV data, which included questions on demographics, climate events, communication, household decision-making and farming practices.

This dataset is expected to contribute to Kenya's ongoing county-level energy planning and provide indicators to better understand local resilience and community needs. The data can provide insights as to how energy services can be strategically planned to strengthen community resilience against existing vulnerabilities and climate change risks. The dataset may also be useful in understanding the role of particular items and appliances in enhancing community resilience and energy security. We hope that the data can be used to provide insights which can help to reduce energy insecurity, improve energy access decisions, and support rural communities’ resilience in both daily life and climate emergencies. Such work can help to mitigate the effects of climate change and support the agricultural sector. This dataset can furthermore help address data gaps pertaining to the impacts of appliances, as outlined in the “Off- and Weak-Grid Appliances Impact Assessment Framework” by Rural Senses et al. [2].

## 3 Data Description

This dataset presents socioeconomic surveys and UPV interviews collected in Siaya County, Kenya. Siaya has undergone significant development over the past decade, particularly in the areas of health and education.

Based on data from the 2019 census [3], Siaya County's population is estimated at 993,183 individuals, with an annual growth rate of 1.7% between 2009 and 2019. The county is comprised of 250,698 households, with an average household size of 3.9. 52.5% of the total population are female, and the majority of residents (91.4%) reside in rural areas. By conducting these surveys and interviews, we hope to support county-level planning in Siaya and in Kenya more broadly. We aim to identify gaps in existing services and infrastructure and provide insight to inform how resources can be allocated to improve overall community well-being. Based on the dataset, we performed a further analysis to identify the household's intersectional needs [4].

The dataset is formed of two parts: (1) household characteristics, obtained from socio-economic surveys; and (2) personal values, gathered through interviews with community members using the UPV game. Surveys and interviews were conducted in six sub-counties of Siaya (i.e., Ugenya, Ugunja, Alego Usonga, Gem, Bondo and Rarieda). They provide insights into the challenges faced by local residents, such as their living situation, infrastructure, household shocks, access to healthcare, education, employment, and subjective well-being.

The dataset is accompanied by a codebook that describes the values that were used in annotation of the UPV game transcriptions and the questionnaires that were used for data collection. All identifying variables, including names, GPS coordinates, and village names were removed, and the dataset is thereby anonymized.

The sample construction process for the dataset is documented in Table 1. A total of 300 household representatives participated in the surveys and interviews. The recruitment venues were specifically selected to try to engage participants from different social classes and with diverse economic needs. To better reflect needs of marginalized groups, we aimed to include a 5% representation of people with disabilities within each sub-county sample. In the final sample, 28 participants (9%) reported disabilities. This strategy helps to ensure that the gathered information can support development efforts that are inclusive and considerate of the needs of all members of the population, particularly those who may be marginalized or disadvantaged.

**Table 1:** Timeline and logistics of dataset construction.

Data Collection	Timeline	2022
	Location	Siaya County
	Collectors	Interviews are conducted by Kenyans fluent in local languages who have been trained through a workshop.
	Methodology	The interviewees were interviewed at the homes of participants. The UPV interviews were conducted in a semi- structured manner, to avoid direct inquiry from the inter- viewer. Additionally, each interviewee completed a socio- economic survey.
Data Translation	Timeline	2023
	Location	Online
	Translators	The translators were Kenyan citizens from neighbouring villages where the data were collected. All translators spoke English fluently as a second language.
Data Annotation	Timeline	2023
	Location	Online
	Annotators	Trained and experienced annotators from primarily Kenya were used to undertake annotation.
	Methodology	We employed trained annotators to conduct sentence-level annotations. Each utterance was carefully annotated with value labels following the UPV game framework. To ensure reliability, each sentence was annotated five times by different annotators. Only labels where a minimum of three out of the five annotators agreed were considered and used. This methodology helps ensure consistency and quality in the annotations.

### 3.1 Survey

The socio-economic survey data was first collected orally in the preferred language of the participants. The collected information was then translated into English. Questionnaire used for the survey are provided as *Questionnaire_Siaya.pdf*.

The results are presented in a CSV file (*Siaya*_*UPV*_*Survey.csv*) where the questions are represented as column headers and the responses of each individual speaker are recorded in the rows. Each row contains a unique interview ID which can be used to inter-relate the data to the other files in this dataset. Note that the CSV file has also been converted to JSON for ease of analysis (*Siaya*_*UPV*_*Survey.json*).

The survey questions were designed to gather data on a wide range of factors that could affect the participants’ perspectives. They fall under the following eight categories: Demographic data, housing status, accessibility of healthcare services, water usage and availability, energy consumption, experience of extreme weather events, community service, and access to information.

**Fig. 1:**
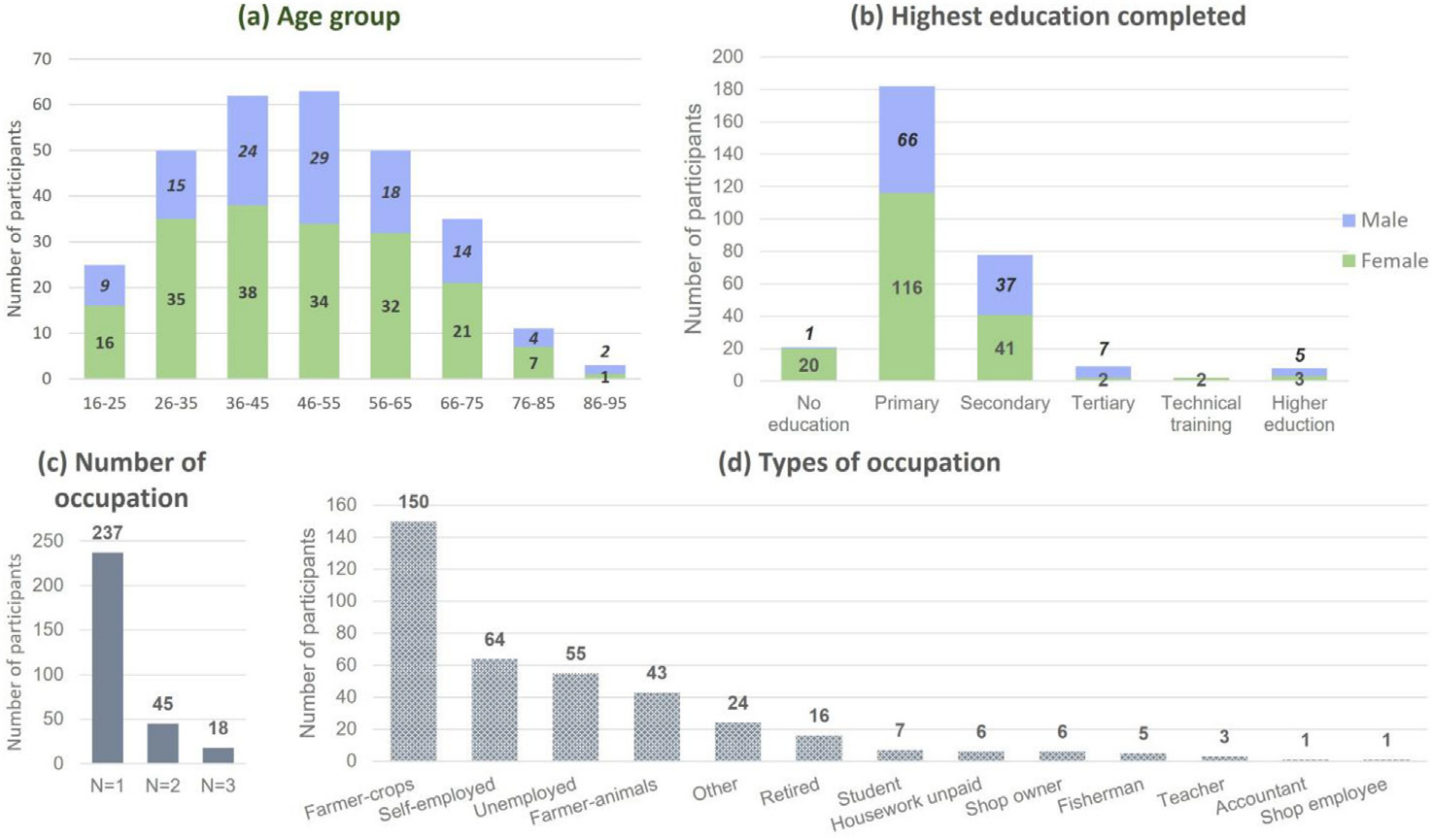
The most concerned extreme climate event perceived by interviewees (*N* = 300).

Fig. 1 illustrates the demographic characteristics of the 300 participants in the study, obtained from the survey data. The sample consisted of 184 females and 116 males, with an age range spanning from 16 to 92 years (Fig. 1a). A greater proportion of females reported no education or primary education only, as indicated by a female/male discrepancy of 20 and 1.76, respectively, which exceeded the overall sample discrepancy of 1.59 (Fig. 1b). Conversely, the trend was inverted for secondary education (1.11), tertiary (0.43), and higher education (0.6), suggesting that males were likely to have higher education opportunities compared to females. 21% of the participants held multiple occupations as shown in Fig. 1c. The largest occupational group among the sample is farmers, comprising 50% (150) of the participants who primarily cultivate crops. Of this group, 14% (43) are livestock farmers, and 35 individuals engage in both crop and animal farming (Fig. 1d).

### 3.2 Interview

The perceived values data, which were derived from the UPV game, are presented in three separate CSV files to facilitate efficient and effective analysis. Note that each CSV file has also been converted to a JSON file with the same name for ease of analysis.

• *Siaya*_*UPV*_*Paragraphs.csv*: During the UPV game, interviewees were asked to select ten items they valued and discuss the reasons behind each selection, which were recorded. In addition, thirteen
• open-ended questions on attitudes and perceptions regarding climate change were asked. Responses to each question were extracted as a separate paragraph. This file therefore extracts each answer to any of these open-ended questions in a separate row (i.e., 3942 rows with one row per paragraph). Table 2 provides a description of the paragraph specifications (i.e., items in the table beginning with P). This dataset also contains interview IDs for interrelation with the survey questions.

**Table 2:** Data specifications in the paragraph- and extract-level UPV datasets. The specifications starting with P are present in both the paragraph and extract file. The specifications starting with E are only present in the extract file.

Key	No.	Value
Paragraph ID	P1	Description: Unique code of each paragraph Type: string
Paragraph Number	P2	Description: Order in which the item was picked by the speaker Type: integer ∈ [1*,* 25]
Questions	P3	Description: Open questions the text is referring to Type: string
Item Name	P4	Description: Item chosen as part of the UPV game Type: string
Text	P5	Description: The answers from interviewee Type: string
Extract ID	E1	Description: Unique code of each utterance Type: string
Start Offset	E2	Description: Locating the first character of each utterance Type: integer
End Offset	E3	Description: Locating the last character of each utterance Type: integer
Sentiment	E4	Description: Type: string ∈ Negative, Neutral, Positive
Annotations	E5	Description: The answers from interviewee Type: string ∈ Faith, Food security, ...…
• *Siaya*_*UPV*_*Utterances.csv*: This file extracts each utterance (i.e., sentence) from the open-ended answers in a separate row (i.e., 16,650 rows with one row per utterance). Additional columns are added to tag the sentiment and value annotations for each sentence, as described in Table 2. This dataset also contains the paragraph specifications and interview IDs for interrelation with the other files. Natural language processing (NLP) methods were employed to extract and analyse the sentences from each paragraph, as discussed by [5].

**Fig. 2:**
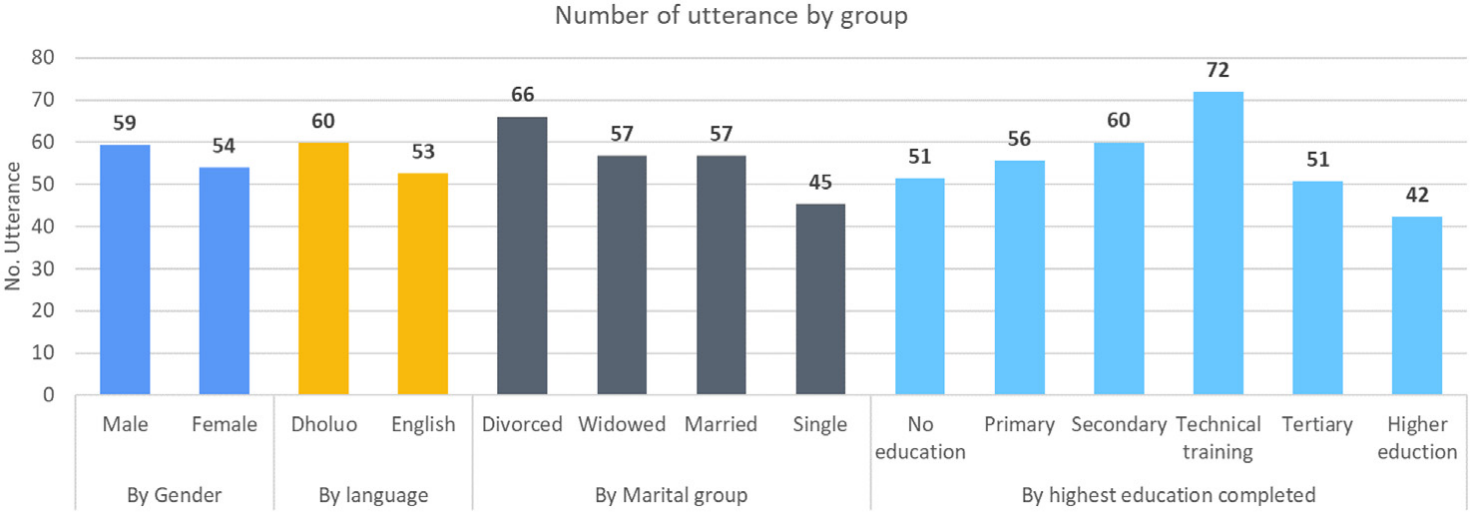
Number of utterances (i.e., sentences) by group throughout the dataset (*N* = 297).

Fig. 2 presents the distribution of utterances based on demographic characteristics. Single females with higher education who respond to questions in English exhibit a lower number of utterances in their answers. Divorced individuals tend to be more talkative when answering questions, whereas singles are generally less talkative in their responses. The impact of education on the number of utterances follows an inverted U pattern, with individuals who have received a moderate level of education exhibiting the highest level of talkativeness. Conversely, individuals with no education and those with higher education use fewer utterances in their responses.

## 4 Experimental Design, Materials and Methods

### 4.1 Case selection

This project took Siaya County in Kenya as a case study. Kenya has made significant economic and political reforms in the past decade that have contributed to sustained economic growth, social development, and political stability. In spite of this, ongoing drought, as well as the increase in the cost of living, have adversely affected households throughout the country. The agricultural sector, which directly contributes 33% to the Gross Domestic Product (GDP) and indirectly contributes an additional 27% to the GDP through linkages with other sectors such as manufacturing and distribution [6], plays a critical role in the country's economy.

Over 40% of the country's population is employed in the agriculture sector, with a significant portion of that employment being in rural areas where 70% of the population is employed in agriculture [7]. Siaya County, located in western Kenya, was selected as a case study due to its high levels of poverty (48% live below the poverty line) and food insecurity. This makes it an interesting location to study climate resilience, as these are risk factors in the case of climate shocks. The agricultural sector, which is particularly important to Siaya's economy and livelihoods, is susceptible to instability due to droughts, floods, etc.

### 4.2 Sampling strategy

The participant selection was guided by the National Commission for Science, Technology, and Innovation (NACOSTI), and was facilitated by the Siaya County Commissioner and local area chiefs. Over a two-week period, 300 individuals from across the county were interviewed. To ensure a diverse representation of the population with varying social and economic backgrounds, a targeted community sampling approach was employed to recruit participants. Participants were selected from four distinct settings: coastal/port towns, urban village towns, rural villages, and special interest villages. Eleven villages, three from each setting, were randomly selected out of the 75 villages in Siaya County [8]. Participants were sourced from diverse socio-economic backgrounds and age ranges, with an aim to achieve a balanced representation of gender, disability, income, age, education, and employment. The sample was intended to represent 5% of people with disability, but the final sample included 9% of those with disabilities. Due to availability and willingness, some demographics were not fully represented, and participants were selected based on their willingness to participate.

### 4.3 Preparation

To ensure the study's integrity, a risk and ethics assessment following the NACOSTI ethics board approval under license No: NACOSTI/P/22/21,652 in research involving human participants was approved. As per the guidance provided by [9], interviewees were compensated for their time. To protect the interviewees’ identities, all names were removed from transcripts in line with NACOSTI ethical procedures.

### 4.4 User perceived value game

The User-Perceived Value (UPV) approach is a data collection method that utilizes a pictorial game-based approach. Participants are asked to select items they value most from a set of 40 everyday items found in case study communities. Among these items, eight are considered to be energy appliances, including a fan, fridge, TV, pump (for irrigation and water), motorbike, pressure cooker, solar PV, and grain mill. The UPV game involves probing the reasoning behind participants’ item selections using 3+ rounds of why-probing process, as previously described by [1,9]. Specifically, the UPV game used in this dataset aims to identify:

1. The five items that communities perceive as important (General format).
2. The appliance that interviewees perceive as most useful and least useful (Appliance-specific format).
3. The three items that are most relevant to the interviewee given the climate event that they are most concerned about. (Climate format).

The full list of survey and UPV questions is provided in Table 3. The UPV questions are numbers 4 to 7.

**Table 3:** Full list of survey questions used in data collection.

Category	No.	Question
Basic information	1	In what language would you prefer to run the interview?
	2	Which of the following assets do you own?
	3	Which of the following items do you have access to in your daily life?
A. Preceived value	4	Which 5 items are most important to you in your daily life? Please indicate these in order of importance, starting with the most important
	5	Which appliance that you perceive as most useful?
	6	Which appliance that you perceive as least useful?
	7	Given the chosen climate event - which 3 items are most useful to you?
B. Demographic	8	Location - sub county
	9	What is your age?
	10	What is your gender?
	11	Marital Status
	12	What is the highest level of education have you completed? [*If Other]
	13	What is your current occupation? [*If Other]
B-1. Household Information	14	Number of females in household
	15	Number of males in household
	16	Who would you define as the head of your household?
	17	Who is the head of the household?
	18	Age of household head?
	19	Household members less than 18
	20	Household members between 18 and 30 years of age
	21	Household members between 31 and 55 years of age
	22	Household members over 55 years of age
	23	Who decides regarding: Buying assets (including animals)
	24	What is the monthly total income of your household (KES)?
B-2. Inclusion	25	Do you have a disability?
	26	What disability do you have?
	27	Type of disability
C. Housing	28	What is the ownership of the dwelling?
	29	Are you happy with the quality of the construction in your house?
	30	What would you change about your house and why?
	31	What are the main 3 building materials used to build your house?
D. Health	32	Who can you consult in case of health issues?
	33	Where do you receive health care treatment?
	34	How long does it take you to travel to receive healthcare?
E. Water	35	Do you treat the water to make it safer for drinking?
	36	What is the use of water from these different sources? [*For each type of usage. Sources including Community tap; Municipality tanker; Your house tap; River; Rainwater harvesting tank; Community well/borehole]
	37	Do you grow your own vegetables?
	38	What vegetables do you grow? [*If Other]
	39	How do you irrigate your land? [*If Other]
	40	How much has your yield increased since you started using an irrigation pump?
F. Electricity	41	What is the main source of lighting?
	42	What is the main source of electricity in your home?
	43	What is the electricity used for?
	44	Do you use electricity for your business?
	45	What do you use electricity for in your business?
	46	What do you use the energy from the following sources for [*For each type of usage. Sources including Coal/Charcoal; Solar (community); Firewood; Solar (private); Other fuels]
G. Extreme weather	47	What extreme climate event are you most worried about?
	48	What other extreme climate events are you worried about?
	49	Why are you most worried about this climate event?
	50	In your own words, how would you describe climate change?
	51	Are you worried about climate change?
	52	What has changed in your community in recent years relating to climate change?
G-1. Flooding	53	Have you ever experienced flooding in your home?
	54	How many times do you experience flooding in your home every year?
	55	In which months have you experienced floods in your home?
	56	Please describe how you were affected by the flooding?
	57	Who would you contact in case of a flood?
	58	Do you have any means of preventing flooding in your home?
	59	Please describe what you would do (actions you take) in case of a flood.
G-2. Fire	60	Have you experienced fire in your home?
	61	(If yes) Please describe how are you were affected by fire?
	62	Who would you contact in case of a fire?
	63	Do you have any means of preventing fire in your home?
	64	Please describe what you would do (actions you take) in case of fire.
G-3. Other hazards	65	Are there any other hazards affecting you and your community?
	66	What other risks or hazards affect you and/or your community?
	67	How many days are spent on average during/after a catastrophic event to return to normality?
	68	How much money [in KES] is spent on average during/after a catastrophic event to return to normality?
H. Community Service	69	Which of the following community services do you use?
	70	How do you get to this community service?
	71	How long does it take to get to this community service?
	72	Do you find it difficult or easy to access this community service?
	73	How do you communicate with other communities?
I. Information communication	74	How do you receive political information? (e.g., about elections)
	75	How do you receive information about health?
	76	How often do you read the newspaper?
	77	How often do you use the internet? (How?)
	78	How often do you watch TV?
	79	How often do you listen to the Radio?
	80	How well connected do you feel within the community?
	81	Generally speaking would say that most people can be trusted or that one can't be too careful in dealing with people?
	82	What community organisation and/or social activity group you are part of? (e.g. church, stokvel, NGO, NPO, Community based organisation, etc.)

### 4.5 Post-processing

The dataset was originally collected in two languages (Dholuo and English) and translated into English by local translators. The data were then extracted into paragraphs and utterances using NLP [5]. The dictionary for the topic's annotation is presented in a python file (*upv*_*definitions.py*).

## Ethics Statements

To ensure the study's integrity, a risk and ethics assessment was undertaken and evaluated by the Medical Sciences Interdivisional Research Ethics Committee (MS IDREC) at the University of Oxford in accordance with the procedures laid down by the University for Ethical Approval for all research involving human participants. It was completed and approved with Reference: R83092/RE001. Additionally, a risk and ethics assessment following the NACOSTI the National Commission for Science, Technology, and Innovation (NACOSTI) ethics board was undertaken, with approval under license No: NACOSTI/P/22/21652 in research involving human participants. To protect the participants’ identity, all names were removed.

## CRediT authorship contribution statement

**Pu Yang:** Investigation, Validation, Writing – original draft, Writing – review & editing. **Alycia Leonard:** Investigation, Validation, Writing – original draft, Writing – review & editing, Supervision. **Micaela Flores Lanza:** Validation, Investigation, Writing – review & editing. **Makena Ireri:** Writing – review & editing. **Stephanie Hirmer:** Funding acquisition, Conceptualization, Writing – review & editing.

## Data Availability

Perceived values and climate change resilience dataset in Siaya County, Kenya (Original data) (Zenodo)
